# Transcriptional and physiological analyses of short-term Iron deficiency response in apple seedlings provide insight into the regulation involved in photosynthesis

**DOI:** 10.1186/s12864-018-4846-z

**Published:** 2018-06-15

**Authors:** Yan-xiu Wang, Ya Hu, Yan-fang Zhu, Abdul Wahid Baloch, Xu-mei Jia, Ai-xia Guo

**Affiliations:** 1 College of Horticulture, Gansu Agricultural University, Lanzhou, Gansu China; 2 Department of Plant Breeding & Genetics, Faculty of Crop Production, Sindh Agriculture University, Tandojam, Pakistan

**Keywords:** RNA-Seq, Iron deficiency, Transcriptome, Photosynthesis, Apple, *Malus halliana*, Chlorophyll

## Abstract

**Background:**

Iron (Fe) is an essential micronutrient for plants. Utilization of Fe deficiency-tolerant rootstock is an effective strategy to prevent Fe deficiency problems in fruit trees production. *Malus halliana* is an apple rootstock that is resistant to Fe deficiency; however, few molecular studies have been conducted on *M. halliana*.

**Results:**

To evaluate short-term molecular response of *M. halliana* leaves under Fe deficiency condition, RNA sequencing (RNA-Seq) analyses were conducted at 0 (T1), 0.5 (T2) and 3 d (T3) after Fe-deficiency stress, and the timepoints were determined with a preliminary physiological experiment. In all, 6907, 5328, and 3593 differentially expressed genes (DEGs) were identified in pairs of T2 vs. T1, T3 vs. T1, and T3 vs. T2. Several of the enriched DEGs were related to heme binding, Fe ion binding, thylakoid membranes, photosystem II, photosynthesis-antenna protein, porphyrin and chlorophyll metabolism and carotenoid biosynthesis under Fe deficiency, which suggests that Fe deficiency mainly affects the photosynthesis of *M. halliana*. Additionally, we found that Fe deficiency induced significant down-regulation in genes involved in photosynthesis at T2 when seedlings were treated with Fe-deficient solution for 0.5 d, indicating that there was a rapid response of *M. halliana* to Fe deficiency. A strong up-regulation of photosynthesis genes was detected at T3, which suggested that *M. halliana* was able to recover photosynthesis after prolonged Fe starvation. A similar expression pattern was found in pigment regulation, including genes for coding chlorophyllide a oxygenase (CAO), β-carotene hydroxylase (β-OHase), zeaxanthin epoxidase (ZEP) and 9-*cis*-epoxycarotenoid dioxygenase (NCED). Our results suggest that pigment regulation plays an important role in the Fe deficiency response. In addition, we verified sixteen genes related to photosynthesis-antenna protein, porphyrin and chlorophyll metabolism and carotenoid biosynthesis pathways using quantitative real-time PCR (qRT-PCR) to ensure the accuracy of transcriptome data. Photosynthetic parameters, Chl fluorescence parameters and the activity of Chlase were also determined.

**Conclusions:**

This study broadly characterizes a molecular mechanism in which pigment and photosynthesis-related regulations play indispensable roles in the response of *M. halliana* to short-term Fe deficiency and provides a basis for future analyses of the key genes involved in the tolerance of Fe deficiency.

**Electronic supplementary material:**

The online version of this article (10.1186/s12864-018-4846-z) contains supplementary material, which is available to authorized users.

## Background

Iron (Fe) is an essential nutrient for plants because of the key role it plays in plant growth and development, especially in respiration, chlorophyll (Chl) biosynthesis and photosynthesis [1, 2]. Although Fe is abundant in the soil, most of it exists in poorly bioavailable inorganic forms that cannot be efficiently absorbed by plants [3]. The obvious effect of Fe deficiency is a pronounced chlorosis due to reduced Chl synthesis [4]. Chl is essential not only as a photosynthetic pigment but also as a structural component in living organisms. The reduced level of chlorophyll molecules decreases the photosynthetic efficiency [5].

Fe deficiency in photosynthetic organisms does not only lead to chlorosis but it is also accompanied by the inhibition of photosynthetic electron transport reactions and by a loss of photosynthetic components and other adverse effects on photosynthesis [6, 7]. Photosynthesis is driven by photosystem I (PSI) and photosystem II (PSII), which are two multisubunit complexes that are embedded in the thylakoid membrane of plants [8, 9]. In the thylakoid membrane, electron transfer processes are initiated by light energy absorbed predominantly by light-harvesting complexes (LHCs). LHCs in plants contain the same pigments, including Chl-a and b molecules as well as a small number of carotenoids associated with the LHCs family of proteins [10, 11]. LHCI and LHCII have been reported to participate in shifts in light-harvesting to energy dissipation [12, 13]. LHCI comprises the four complexes Lhca1 to 4, which naturally assemble into the heterodimers Lhca1/4 and Lhca2/3 [14, 15]. In addition, two minor LHCI-like proteins (Lhca5 and 6) that have a high degree of similarity to Lhca1 to 4 were identified in *Arabidopsis* [10]. Cross-linking studies indicated that Lhca5 interacts with LHCI in the Lhca2/3 heterodimer [16]. Additionally, Lhca5 and Lhca6 were shown to be associated with efficient operation of NAD(P)H dehydrogenase [17]. LHCII is a trimer of any combination of the three complexes Lhcb1 to 3 with a high degree of similarity, yet the minor antenna consists of the three monomers from Lhcb4 to 6. Lhcb3 to 6 are exclusively associated with PS II and Lhcb1 and 2 form mixed trimers that can be associated with either photosystem.

**Fig. 1:**
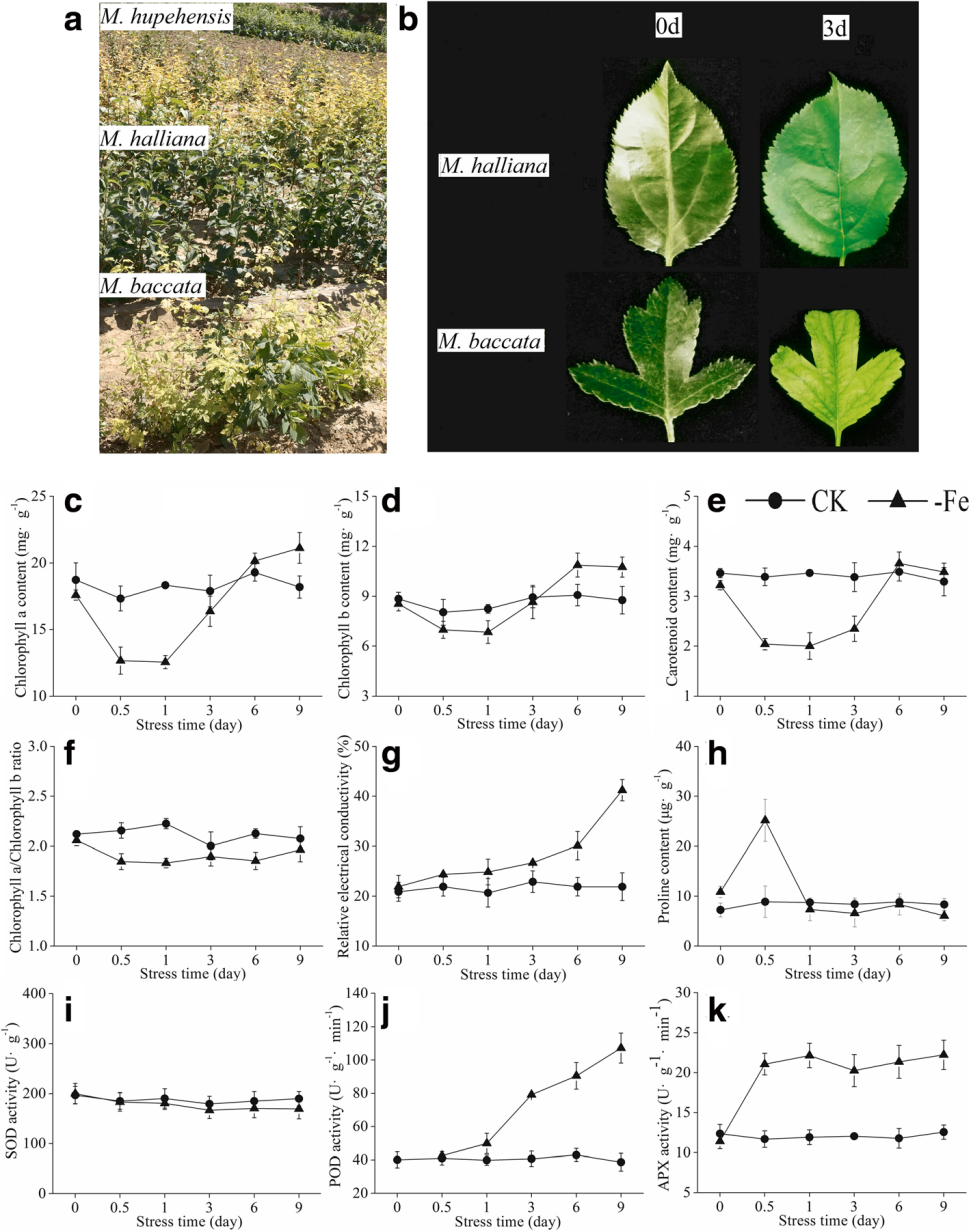
**a** The field performances of *M. hupehensis*, *M. halliana* and *M. baccata* on the Loess Plateau in the Northwest of China. **b** Hydroponic seedlings performances of *M. halliana* and *M. baccata* under Fe deficient stress. Physiological parameters: (**c**) chlorophyll a content, (**d**) chlorophyll b content, (**e**) carotenoid content, (**f**) chlorophyll a/chlorophyll b ratio, (**g**) relative electrical conductivity, (**h**) proline content, (**i)** superoxide dismutase activity, (**j**) peroxidase activity and (**k**) ascorbateperoxidase activity of *M. halliana* under Fe-deficiency (-Fe) and Fe-sufficiency (CK)

In the Northwest Loess Plateau region, Fe deficiency is one of the major problems limiting the yield and quality of fruit. Under high pH and CaCO_3_ soil conditions, applying Fe into soil may not solve the problem [18]. Therefore, selecting Fe deficiency-tolerant rootstocks can be an effective and environmentally friendly strategy to minimize Fe-deficiency problems in the apple industry. *Malus halliana* is an indigenous plant cultivar belonging to the *Malus* genus in the Rosaceae family. We have found that *M. halliana* grows well and the chlorotic symptom associated with Fe deficiency in apple trees was not found on the cultivar in the Northwest Loess Plateau of China (Fig. 1a). *M. halliana* showed characteristics of Fe deficiency-tolerant rootstocks. In this study, we measured physiological, photosynthetic and fluorescence parameters, and also used RNA-Seq technology to explore the transcriptional changes in *M. halliana* leaves subjected to Fe-deficient stress to investigate the possible molecular mechanisms of Fe deficiency tolerance in the cultivar.

## Results

### Selection of timepoints for transcriptome analysis

To determine suitable timepoints for transcriptome analysis, we investigated the phenotypic changes in *M. halliana* and *M. baccata* and measured the physiological parameters of *M. halliana* under Fe deficiency stress. Under hydroponic conditions, slight chlorosis in new leaves of *M. baccata* was found after 3 d exposure to Fe deficiency, whereas there was no chlorosis in *M. halliana* (Fig. 1b). In fact, the pigment content of *M. halliana* was changed under Fe-deficiency stress. Chl a, Chl b and carotenoids decreased at 0.5 and 1 d; however, they increased at 3, 6 and 9 d (Fig. 1c–e). Compared to the control, the ratio of Chl a/b decreased after Fe deficiency treatment (Fig. 1f). The relative electrical conductivity (REC) of *M. halliana* seedlings increased gradually and reached 41.20% at 9 d (Fig. 1g). Except for the 0.5 d timepoint, there were no significant differences in proline (Pro) content between the treated group and the control group at other timepoints (Fig. 1h). At 0.5 d, the Pro content of the treatment group increased rapidly and reached 25.15 μg·g^− 1^, which was 2.83-fold higher than the amount found in the control. Superoxide dismutase (SOD) did not respond to Fe deficiency and showed little change in SOD activity (Fig. 1i). Peroxidase (POD) activity increased with the extension of stress time (Fig. 1j). POD activity increased rapidly at 3 d by 90.74% over the activity at 0 d. Ascorbate peroxidase (APX) activity increased at 0.5 d and maintained this level of activity under subsequent stress (Fig. 1k). Based on these results, we sequenced the transcriptome of *M. halliana* for 0 d (T1), 0.5 d (T2) and 3 d (T3) under Fe deficiency stress.

### RNA-Seq transcriptome of *M. halliana*

To understand the molecular basis of Fe-deficiency tolerance in *M. halliana*, RNA-seq libraries were established from leaves of Fe-deficiency treated seedlings at three timepoints. The correlation among gene expression levels in samples is an important index for the reliability test of experiments. In this study, the correlation coefficients were more than 0.95 (Additional file 1). RNA-Seq generated more than 34.89 million raw reads for each sample, and two biological replicates were set for each timepoint (Table 1). Of these reads, GC content was approximately 47.00% for the libraries. After quality control, 33.34 to 44.14 million clean reads were yielded with more than 97.09% Q20, and 24.28 to 31.99 million clean reads were mapped to the apple genome.

**Table 1:** Summary of transcriptome sequencing data from leaves of *M. halliana* under three Fe deficiency timepoints

Samples	Raw Reads	Clean Reads	Mapped Reads	Q20 (%)	GC content (%)
T1_rep1	38,249,468	37,405,176	26,445,784 (70.7%)	97.09	47.16
T1_rep2	37,637,288	36,841,980	25,785,714 (69.99%)	97.18	46.95
T2_rep1	46,148,652	44,139,498	31,987,790 (72.47%)	97.16	46.89
T2_rep2	36,774,908	35,150,068	25,248,424 (71.83%)	97.51	46.99
T3_rep1	42,471,554	40,785,670	29,900,697 (73.31%)	97.53	47.43
T3_rep2	34,886,520	33,339,474	24,275,046 (72.81%)	97.61	47.05

Q20% is the proportion of the nucleotide quality value larger than 20; GC content is proportion of guanidine and cytosine nucleotides among the total nucleotides

### Differentially expressed genes (DEGs) under iron deficiency

**Fig. 2:**
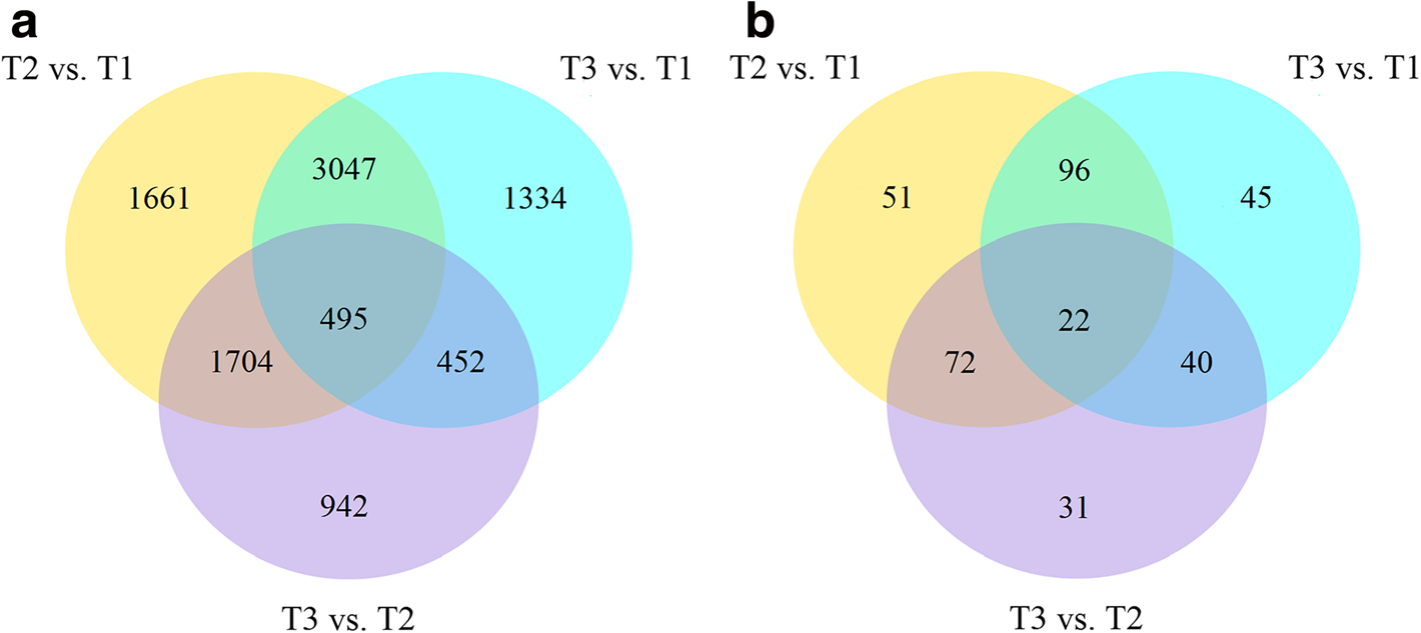
**a** Venn diagram of DEGs at three timepoints, (**b**) Venn diagram of DEGs involved in photosynthesis at three timepoints

Differences in gene expression at three timepoints under Fe deficiency were examined with an adjusted *P*-value (p-adj) < 0.005 and |log2(Fold Change)| > 1 as the threshold, and DEGs were identified by 3 pair-wise comparisons (Fig. 2a). We found 6907, 5328, and 3593 DEGs in pairs of T2 vs. T1, T3 vs. T1, and T3 vs. T2. Comparisons of these three datasets showed that 495 genes overlapped among T2 vs. T1, T3 vs. T1, and T3 vs. T2.

### Functional classification of DEGs under iron deficiency

To deepen our understanding of the functions of these DEGs, Gene ontology (GO) term enrichment analysis was performed (Additional file 2). More than 42 significantly enriched GO biological terms were enriched, including protein phosphorylation, the cellular protein modification process, the protein modification process, phosphorylation and the phosphate-containing compound metabolic process. The cellular component categories of DEGs were significantly enriched for the oxidoreductase complex, ubiquitin ligase complex, apoplast, thylakoid membrane and photosystem II oxygen evolving complex. Significantly enriched GO molecular terms included protein kinase activity, phosphotransferase activity, tetrapyrrole binding, heme binding and iron ion binding.

**Fig. 3:**
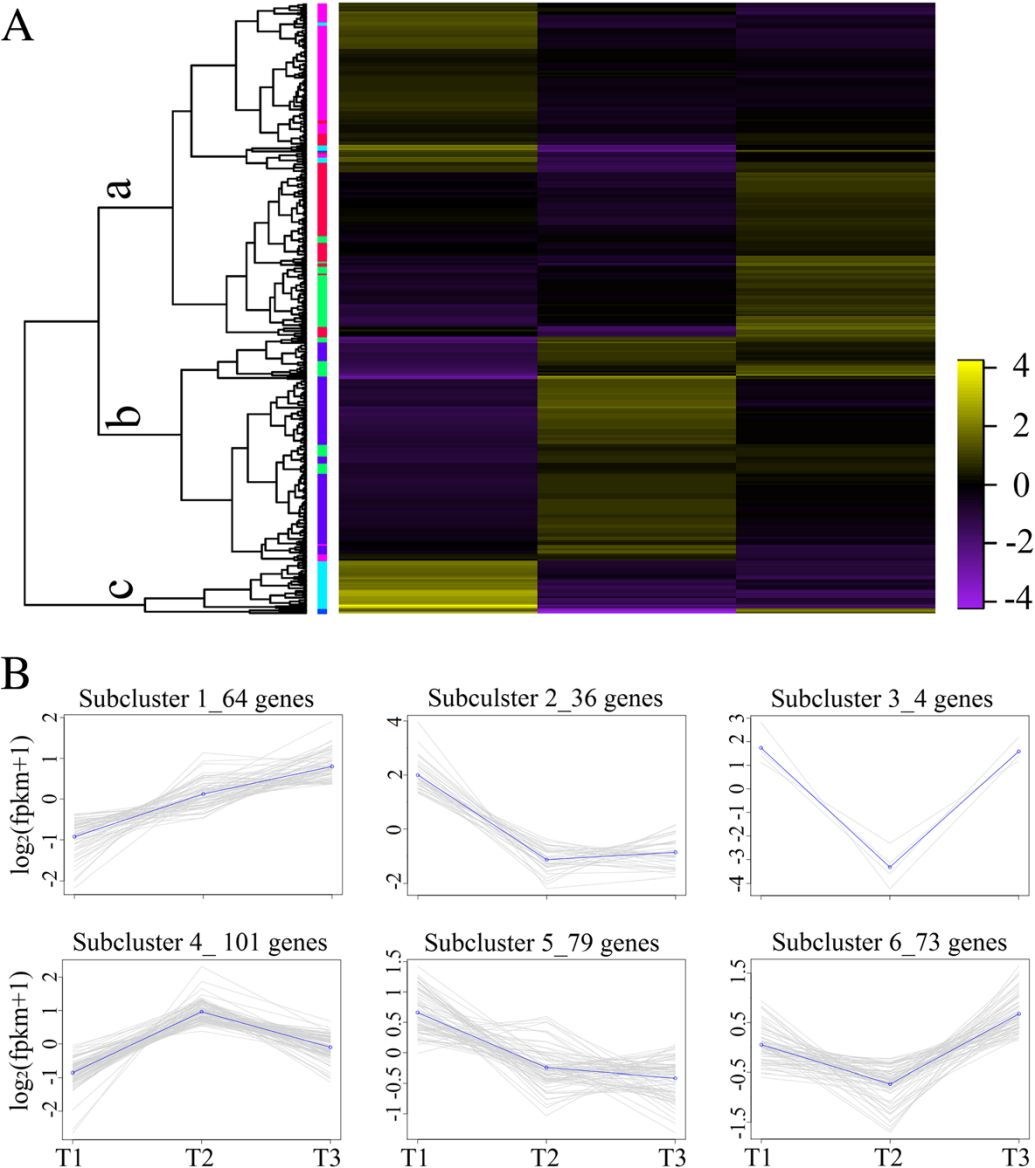
**a** Heat map analysis showing the photosynthesis related gene expression patterns during the iron-deficiency process. Each gene at each of the three timepoints was represented as a horizontal short line. The different colors of the band on the right side of the dendrogram represent different subclusters. **b** Gene expression patterns of subclusters

We focused our attention on photosynthesis-related terms, including iron binding, heme binding, photosystem II, thylakoid, the Chl catabolic process, the Chl biosynthetic process, ATP synthesis-coupled electron transport and the carotenoid biosynthetic process. A total of 357 DEGs involved in photosynthesis were screened. We found 241, 203 and 165 photosynthesis-related DEGs in T2 vs. T1, T3 vs. T1, and T3 vs. T2, respectively. A comparison of these three datasets showed that 22 genes overlapped (Fig. 2b). According to a dendrogram, 357 genes were divided into three distinct cluster groups, including a, b and c. Group a included 195 genes that were down-regulated at T2, Group b included 131 genes that were up-regulated, and Group c included 31 genes that showed a decreasing trend (Fig. 3a). To understand the expression patterns of photosynthesis-related genes over the three timepoints, 357 genes were sorted into six subclusters to gain insight into the genomic reprogramming of Fe-deficiency treatment (Fig. 2b). Subcluster 1 containing 64 genes showed elevated expression among three timepoints. Gene expression in subclusters 2, 3 and 6 decreased at T2 while expression in subcluster 4 increased. Subcluster 5 contained 79 genes and showed an inverse expression pattern to subcluster 1 (Fig. 3b). Subcluster 1 contained 31 cytochrome genes, 15 enzyme genes, 7 protein genes, 6 photosystem genes and 5 other genes, which were mainly involved in photosynthesis and metabolic pathways. Subcluster 2 contained 19 enzyme genes, 9 protein genes, 5 cytochrome genes and 3 uncharacterized genes. Subcluster 3 contained only 4 genes including 2 cytochrome genes and 2 photosystem genes. There were 38 enzyme genes, 36 protein genes, 10 cytochrome genes, 4 subunit genes and 13 other genes found in subcluster 4. Additionally, 43 enzyme genes, 18 protein genes, 9 cytochrome genes and 9 other genes in subcluster 5. Subcluster 6 contained 30 enzyme genes, 22 protein genes, 14 cytochrome genes and 7other genes (Additional file 3).

### DEGs related to photosynthesis

**Fig. 4:**
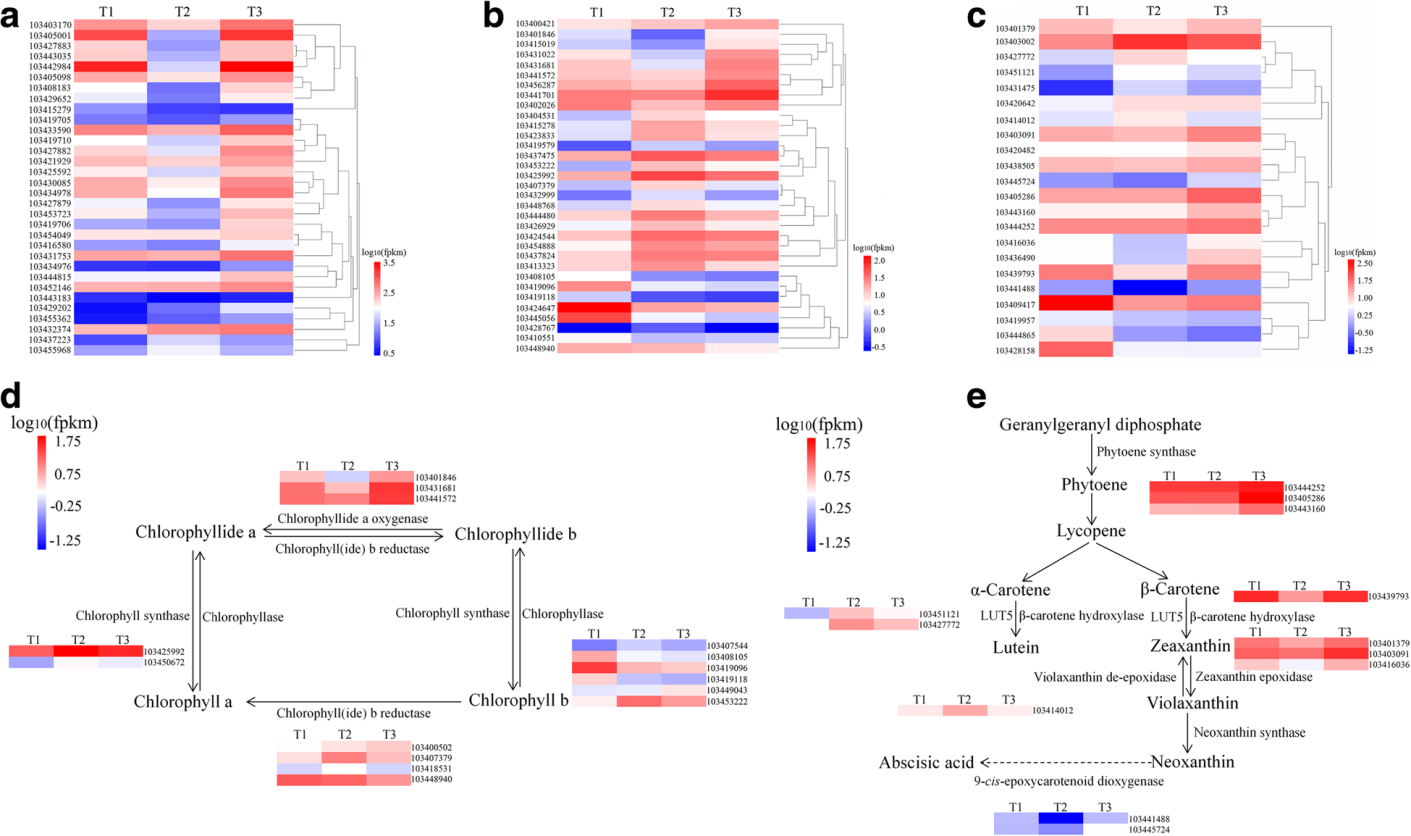
**a** Heat map analysis showing the gene expression patterns involved in photosynthesis-antenna protein pathway during the iron-deficiency process. **b** Heat map analysis showing the gene expression patterns involved in porphyrin and chlorophyll metabolism pathway during the iron-deficient-process. **c** Heat map analysis showing the gene expression patterns involved in carotenoid biosynthesis pathway during the iron-deficiency process. **d** Partial porphyrin and chlorophyll metabolism pathway that differentially expressed genes involved in during the iron-deficiency process. **e** Partial carotenoid biosynthesis pathway that differentially expressed genes involved in during the iron-deficiency process. Each gene at each of the three timepoints was represented as a horizontal short line

To decipher the functions of DEGs, we mapped these genes in the Kyoto encyclopedia of genes and genomes (KEGG) database. The DEGs were associated with various KEGG pathways involved in photosynthesis, signal transduction and metabolism-related pathways (Additional file 4). Notably, we observed specific enrichment of genes in the photosynthesis-antenna proteins pathway (mdm00196), porphyrin and Chl metabolism (mdm00860) and carotenoid biosynthesis (mdm00906). The expression levels of selected the thirty two genes coding antenna proteins identified from KEGG pathway analysis are shown in Fig. 4a.

Many genes assigned to photosynthesis-related pathways, such as photosynthesis-antenna protein, porphyrin and Chl metabolism as well as carotenoid biosynthesis were identified (Fig. 4). Most genes involving the photosynthesis-antenna protein pathway showed decreased expression patterns at T2 (Fig. 4a). Porphyrin and Chl metabolism-related genes were divided into three groups. The first group and the second group were enriched in genes that were up-regulated at T3 and T2, respectively. The other group containing eight genes showed consistent down-regulation (Fig. 4b). The genes involved in the carotenoid biosynthesis pathway were divided into two groups that showed opposite expression patterns (Fig. 4c). One group contained genes that were up-regulated at T2, and the other groups were down-regulated.

Chlorophyllide a oxygenase (CAO), chlorophyll (ide) b reductase (CBR), Chl synthase (CS) and Chlase played important roles in the transformation between Chl a and b. Three genes encoding the CAO showed a falling-rising expression pattern under Fe-deficiency. Gene expression of most *CBR* decreased at T3. *CS* genes were up-regulated at T2 and down-regulated at T3. Five Chlase genes were down-regulated at T3 and 1 gene was up-regulated (Fig. 4d).

Carotenoid biosynthesis starts with the condensation of two geranylgeranyl pyrophosphate (GGPP) molecules by phytoene synthase (PSY) to form phytoene. *PSY* genes were up-regulated at T3, whereas there were no obvious changes at T2. The genes coding for β-carotene hydroxylase (β-OHase), zeaxanthin epoxidase (ZEP) and 9-*cis*-epoxycarotenoid dioxygenase (NCED) were down-regulated at T2 and up-regulated at T3. In contrast, *LUT5* and violaxanthin de-epoxidase (*VDE*) genes were up-regulated at T2 and down-regulated at T3 (Fig. 4e).

### Verification of DEG analysis results by qRT-PCR

**Fig. 5:**
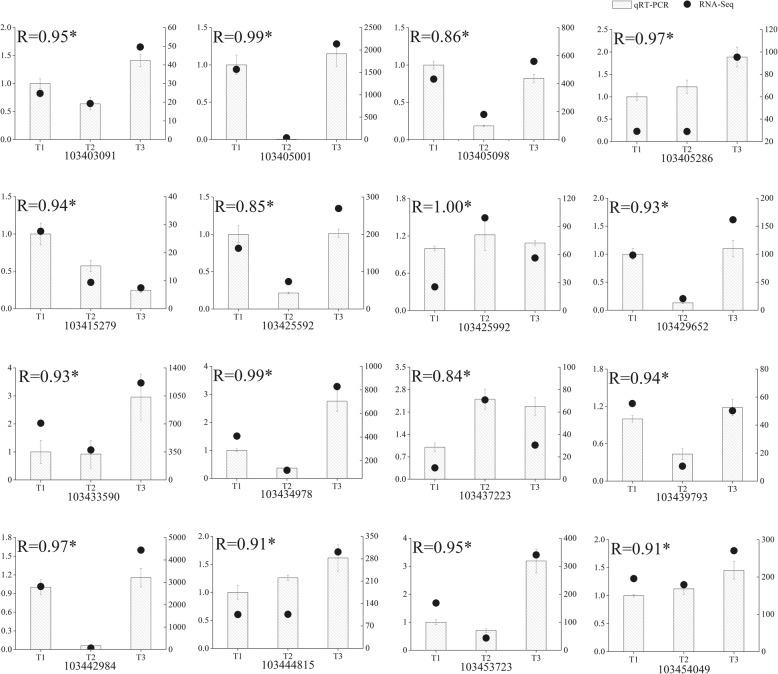
qRT-PCR confirmation of sixteen candidate genes at three timepoints: T1, T2 and T3

To validate the results of DEG analysis, the relative expression levels of sixteen genes involved in photosynthesis-antenna protein, porphyrin and chlorophyll metabolism and carotenoid biosynthesis pathways were measured by quantitative real-time PCR (qRT-PCR) using independent samples with the same treatments used for the RNA-Seq analysis. Measurements were replicated three times. The qRT-PCR primer pairs are listed in Additional file 5. All genes showed significant correlations (*P* = 0.05) between the qRT-PCR results and the RNA-Seq analysis, which indicated that the RNA-Seq data were highly reliable (Fig. 5).

### Quantitative analysis of chlorophyll fluorescence, photosynthetic parameters and chlorophyllase activity

**Fig. 6:**
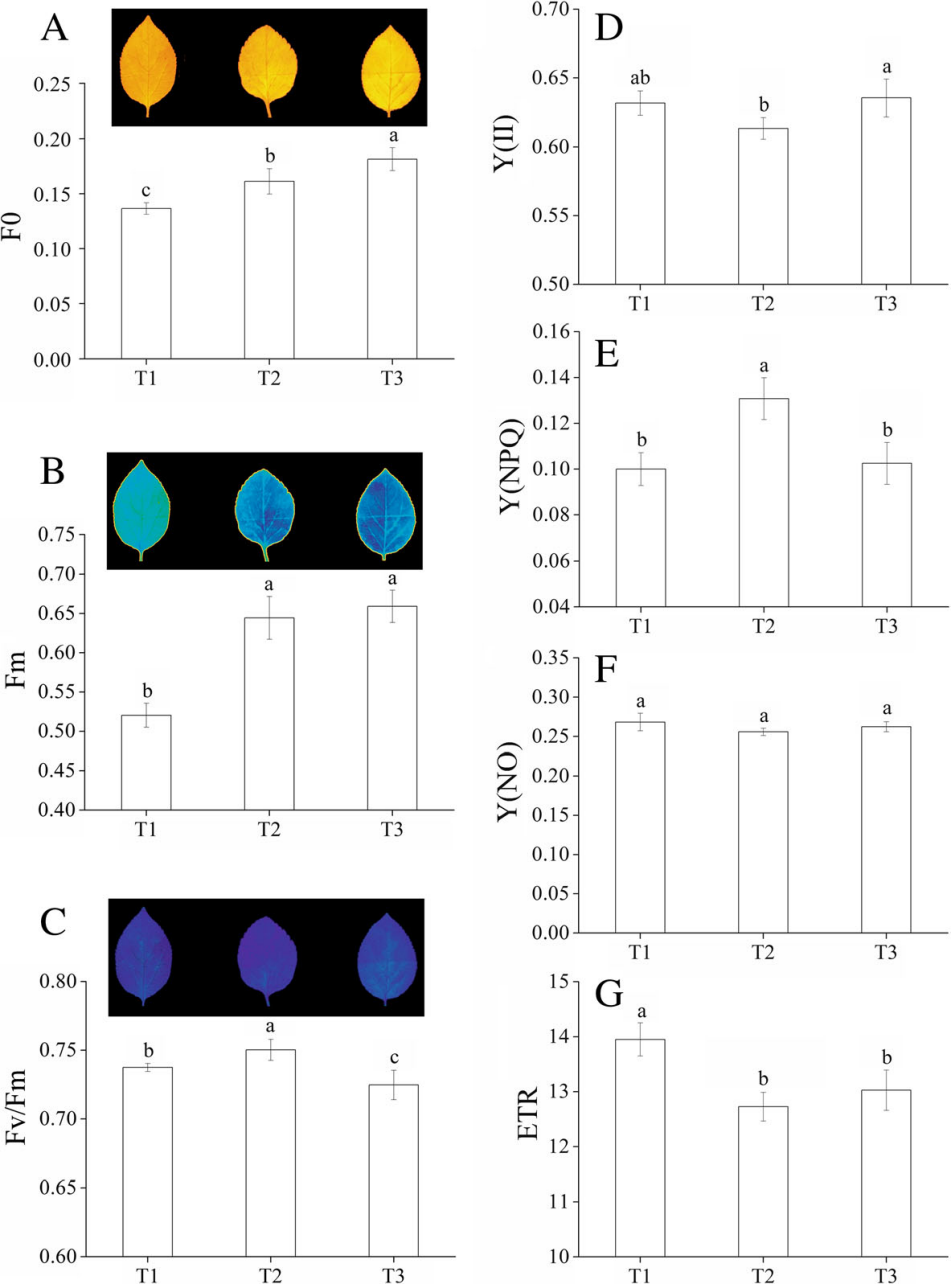
Photosynthetic fluorescence parameters of *M. halliana*. Images and values of (**a**) minimum fluorescence (F0), (**b**) maximum fluorescence (Fm) and (**c**) maximum quantum yield (Fv/Fm) and values of (**d**) effective quantum yields (Y(II)), (**e**) quantum yield of regulated energy dissipation (Y(NPQ)), (**f**) quantum yield of non-regulated energy dissipation (Y(NO)) and (**g**) relative electron transport rates (ETR) of *M. halliana* during the iron-deficiency process

**Fig. 7:**
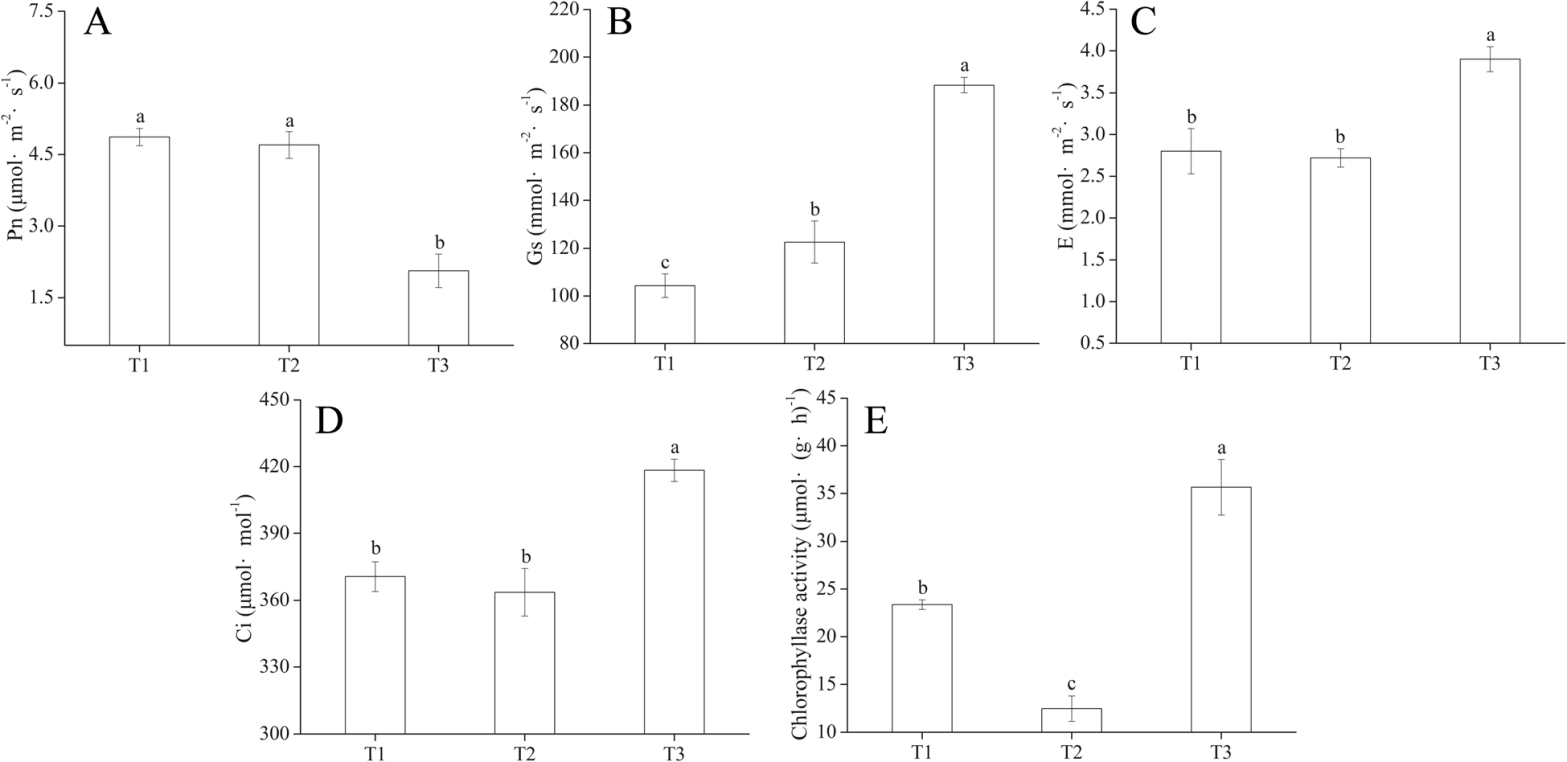
Photosynthetic parameters of *M. halliana*. **a** Net photosynthetic rate (Pn), (**b**) Stomatal conductance (Gs), (**c**) Transpiration rate (**e**) and (**d**) Intercellular CO_2_ concentration (Ci) of *M. halliana* during the iron-deficiency process. **e** Chlorophyllase activity of *M. halliana* under T1, T2 and T3

We measured Chl fluorescence parameters with an Imaging-Maxi-PAM device (Fig. 6). The minimum fluorescence (F0) and maximum fluorescence (Fm) values at T1 were significantly lower compared to values at T2 and T3 (Fig. 6a, b). We found significant differences in maximum quantum yield (Fv/Fm) among T1, T2 and T3 (Fig. 6c). There was a significant difference between T2 and T3 in effective quantum yields (Y(II)) (Fig. 6d). The quantum yield of regulated energy dissipation (Y(NPQ)) at T2 was significantly higher than that of T1 and T3 (Fig. 6e). The quantum yield of non-regulated energy dissipation (Y(NO)) was not significantly different among the three timepoints (Fig. 6f). *M. halliana* had higher relative electron transport rates (ETR) without Fe deficiency stress (Fig. 6g). Photosynthetic characteristics of *M. halliana* were measured under low Fe stress. Net photosynthetic rate (Pn) decreased at T3, but stomatal conductance (Gs), transpiration rate (E) and intercellular CO_2_ concentration (Ci) had higher values at T3 (Fig. 7a–d). Chlase activity decreased at T2 but increased at T3 (Fig. 7e). The activity at T3 was 1.86 and 0.53-fold higher compared to T2 and T1, respectively.

## Discussion

Fe deficiency is a serious problem for organisms, especially for photosynthetic organisms [12] because it is a cofactor in a photosynthetic apparatus, such as PSII, PSI and the cytochrome b6/f complex [19]. To study molecular changes under Fe deficiency, we sequenced the transcriptome of *M. halliana* at three timepoints. The results of the transcriptomic analyses showed that *M. halliana* was able to withstand short-term of Fe-deficiency. The tolerance to Fe stress was supported by photosynthetic recovery. Simultaneously, pigment regulation is coordinated with photosynthetic responses. The regulation of overall metabolic pathways seems to be the basis of the ability to overcome short-term Fe-deficiency stress by the cultivar. We present a comprehensive mechanism of how *M. halliana* resists to Fe deficiency in multiple ways, including physiology, transcription, genetics, photosynthesis and fluorescence.

Chl molecules play central roles in photosynthesis because they are capable of harvesting light energy and driving electron transfer [20]. The newly formed Chl binds to proteins to form Chl-protein complexes during greening [21]. Chl-protein complexes may be classified in two groups: Chl a-protein complexes, which form the core antennae of photosystems and LHCs which form antennae of PSI and PSII [9, 10]. Chl synthesis is finely regulated to supply the Chl required for the formation of photosystems. Under Fe stress, the content of Chl a and b as well as the ratio of Chla/b in *M. halliana* decreased at T2 and increased at T3 (Fig. 1a, b, d). This change may be a way for *M. halliana* to respond to Fe-deficiency stress by regulating pigment contents and proportions. Conversion can occur between Chl a and b, and the first step of conversion is catalyzed by CBR [22]. Four genes coding for CBR appeared to have different varieties in transcription levels (Fig. 4d). From the expression of *CAO* genes, we believe the expression of *CBR* genes increased at T2 and decreased at T3. The close relationship between chlorophyll synthesis and LHC formation has clarified that Chl b is required to form LHCs [23]. Webb and Melis [24] showed that *CAO* and *Lhcb* transcripts coincident with a period of rapid LHC apoprotein accumulation. Masuda et al. [25] suggests that the *CAO* gene expression is coordinated with the *Lhcb* gene. In this experiment, the change in CAO genes was consistent with that in *Lhcb* genes, excluding *Lhcb4* genes. Although the Chl a and b contents decreased at T2, the *CS* genes showed high expression at T2 (Fig. 4d). Additionally, the genes coding for Chlase showed different trends under Fe-deficiency conditions, whereas the activity of Chlase decreased at T2 and increased at T3, which differed from any of the Chlase genes (Fig. 7e), and suggests that Chlase is encoded by multiple genes and is subject to post-transcriptional regulation under Fe deficiency. The increase in *CS* transcription and the decrease in Chlase activity at T2 suggested that *M. halliana* tried to increase Chl content to resist Fe deficiency stress.

Similar regulation was also observed for carotenoid biosynthesis. Carotenoids are the second most abundant pigments in nature. Carotenoids and their oxidative and enzymatic cleavage products have functional roles in various biological processes in plants, such as the assembly of photosystems and light harvesting antenna complexes for photosynthesis and photoprotection [26, 27]. PSY, which is the first step in the carotenoid biosynthesis, is considered a rate-limiting step in carotenoid biosynthesis [28]. In response to various factors, such as development, photoperiods, abiotic stresses and post-transcriptional feedback regulation, *PSY* genes are induced in transcription [26]. In this experiment, *PSY* genes changed slightly at T2 but showed high transcription at T3, indicating that *PSY* responded to Fe deficiency stress at T3 (Fig. 4e). Interestingly, the carotenoid content decreased at T2 and increased at T3 (Fig. 1e). Although we know about the core carotenoid enzyme, our understanding of carotenoid biosynthesis under Fe-deficiency stress is still limited, which is partially due to the diversity and complexity of products and enzymes. Typically, β-carotene, lutein and violaxanthin are accumulated in leaves. Enzymes responsible for the formation of these products, including PSY, β-OHase, ZEP and VDE, have different variation patterns at the transcript level. *β-OHase* and *ZEP* genes were down-regulated at T2 and up-regulated at T3. Expression of *VDE* genes and that of *β-OHase* as well as *ZEP* were opposite (Fig. 4e), which explains the possible causes of changes in carotenoid content. As the substance that promotes thermal dissipation in LHCs, Zeaxanthin plays a critical role in photoprotection [29]. Zeaxanthin is linearly related to NPQ. Our data showed that Y (NPQ) increased at T2 and decreased at T3, which may indicate the amount of zeaxanthin content in the cell (Fig. 6e). However, *β-OHase* showed conflicting results at the transcript level. *β-OHase* post-transcriptional or translational control might be a more decisive factor in the regulation of *VDE* [30]. In addition, zeaxanthin can be synthesized from antheraxanthin via the xanthophyll cycle [31]. Bouvier et al. [32] suggest that reduced ferredoxin availability may limit zeaxanthin epoxidation. The decrease in *ZEP* transcription might be a rapid response to Fe deficiency stress.

In higher plants, a decrease in photosynthetic protein abundance, electron transport chain components and the quantum yield of PSII are caused by Fe deficiency [33–35]. The LHCs play an important role in absorbing light and transferring energy to the center of the photosystem. The formation of LHCs will change to some degree due to the difficulty in Chl biosynthesis under Fe deficiency [36]. Transcriptionally, most *Lhc* genes decreased at T2 and were elevated at T3 (Fig. 4a). For heterodimers Lhca1/4 and Lhca2/3, the transcriptional expression between *Lhca1* and *4*, and between *Lhca2* and *3* was closely related. Transcripts of *Lhca*1 and 4 showed a gradual rise and fall, respectively. *Lhca2* and *3* were down-regulated and up-regulated expressed at T2 and T3, respectively. Compared to other *Lhca* genes, *Lhca5* was specifically up-regulated at T2 and down-regulated at T3 (Fig. 5). Consistently, the results for Ganeteg et al. [37] indicate that *Lhca5* seems to be regulated differently from other LHC proteins since *Lhca5* mRNA levels increase under high light conditions. Furthermore, *Lhca5* interacts in a direct physical manner with LHCI in *Lhca2* or *Lhca3* [16, 37]. This experiment also illustrates this point because the expression pattern of *Lhca5* is negatively related to *Lhca2* and *3*. Except for *Lhcb4*, other *Lhcb* genes were down-regulated at T2 and up-regulated at T3 (Fig. 5). The *Lhcb4* gene product may involve processes of energy balancing or dissipation in higher plants [38]. This finding reveals why the *Lhca4* is different, which is related to the xanthophyll cycle and thermal dissipation. The results, therefore, suggest that Fe deficiency regulates light harvesting to affect photosynthesis, which may be an adaptive mechanism of *M. halliana* to Fe deficient stress.

We compared the PSII photosynthetic activities among three timepoints with Chl fluorescence parameters. *M. halliana* had significantly higher Fv/Fm at T2 compared to of T1 and T3, which suggests that PSII activity at T2 was higher than at T1 and T3. The significant decrease in photosynthetic electron transport was found in Fe-deficient grapevine leaves [39]. In our experiments, ETR reduction was also observed (Fig. 6g). The increase of F0 indicated that the photosynthetic apparatus was gradually damaged under Fe deficiency stress. Fe deficiency induced a significant decrease in Pn, whereas the Gs remained balanced, which suggested that a decline in Pn could not be attributed to stomatal factors [40]. However, our data showed that under the Fe deficiency, Ci increased at T3, Gs gradually increased, and Pn decreased at T3 (Fig. 7a, b, d). These data implied that the decrease in the Pn had been caused not only by stomatal limitations but also by non-stomatal limitations. Additionally, the decrease in the Pn at T3 implicated that the response of photosynthetic recovery is later than the recovery of pigment contents. As for some genes related to photosynthesis, such as *Lhc*s, although the gene expression levels at T3 are increased, the photosynthetic response had not been fully stimulated which could be due to short response time.

## Conclusions

*M. halliana* can overcome short-term Fe deficiency by photosynthetic recovery. Furthermore, pigment regulation is also an important part of stress responses, and is coordinated with photosynthetic responses. This study provides a solid foundation for improved understanding of Fe tolerance responses in apples and also gives insights into the characterization of Fe resistance genes. In future studies, we will focus on investigating the process of chlorophyll biosynthesis and metabolism under Fe deficiency stress as well as screening for key genes for cloning and functional analysis.

## Methods

### Plant materials

Seeds of *M. halliana* were obtained from the Gansu province of China, and were surface sterilized in 0.2% KMnO4 for 30 min, then washed with running water for 12 h. The seeds were subsequently stored in low temperature sand for 35 d and later sown in small nutritional pots. Seedlings with 6 true leaves were selected and transferred to foam boxes containing half-strength Han’s nutrient solution [41]. Five seedlings were grown in each box, and all plants were exposed to a uniform growth environment. The nutrient solution was aerated continually and renewed every 7 d. After 20 d, the uniform seedlings were selected and subjected to carry out treatments of two Fe levels: CK (40 μmol·L^− 1^) and -Fe (4 μmol·L^− 1^).

### Determination of physiological parameters

Chl a, Chl b, the Chl a/b ratio and carotenoid contents were determined from absorption spectra using Arnon’s method [42]. REC was determined by a conductance meter (DDS-307) (LeiCi, Shanghai, China) as described by Zhao [43]. Pro content was determined with the ninhydrin colorimetry method [44]. The activity of SOD, POD and APX were assayed as described by Hameed [45].

### Transcriptome sequencing

To gain insight into the molecular mechanisms responsible for regulation in low Fe conditions of *M. halliana*, transcriptomic analysis was carried out using leaf samples of *M. halliana*. To ensure data reliability, two replicates of each sample were sequenced. Euphylla samples were harvested and immediately immersed in liquid nitrogen. Leaves from 10 individuals grown under Fe-deficiency conditions were pooled as biological replicates for RNA extraction. Total RNA was isolated using a TRIzol kit (Invitrogen, Carlsbad, CA, USA). Sequencing libraries were generated using the NEBNext® UltraTM RNA Library Prep Kit for an Illumina® device (NEB, Ipswich, MA, USA). Clustering of the index-coded samples was performed on a cBot Cluster Generation System using the TruSeq PE Cluster kit v3-cBot-HS (Illumina). After cluster generation, library preparations were sequenced on an Illumina Hiseq 4000 platform. Clean data were obtained by removing reads containing the adapter, reads containing ploy-N and low-quality reads from the raw data. The abundance values for all the genes were normalized and calculated (using uniquely mapped reads) by the expected number of fragments per kilobase of transcript sequence per million base pairs sequenced (FPKM) method. Differential expression in paired samples was screened using a DEG-seq method (adjusted *P*-value (*p*-value) < 0.005 and |log2(FoldChange)| > 1). The DEGs that were identified were subjected to GO and KEGG pathway enrichment analyses using GO-SEQ and KOBAS 2.0, respectively. GO enrichment analyses were based on the Wallenius noncentral hypergeometric distribution (corrected *P*-value ≤ 0.005). KEGG pathway enrichment analyses with a parameter setting of false discovery rate (FDR) ≤ 0.05 were conducted.

### Quantitative real-time PCR

cDNA was synthesized from total RNA using the PrimeScript™ RT reagent Kit with gDNA Eraser (Perfect Real Time) (TaKaRa, Dalian, China). Quantitative real-time PCR was performed using a Light Cycler® 96 Instrument (Roche, Shanghai, China) with *GAPDH* as a reference gene. Measurements for each plate were replicated three times. Real-time PCR primer pairs are listed in Additional file 5.

### Chlorophyll fluorescence parameters and photosynthetic parameters

Chl fluorescence parameters were measured using an Imaging-PAM-MAXI instrument (Walz, Germany). Photosynthetic characteristics of leaves at different timepoints were determined by a CIRAS-2 photosynthetic instrument (PP-System, UK). Before determining the characteristics, a sulfur lamp was used to induce photosynthesis for half an hour. Chlorophyllase (Chlase) activity was measured and calculated according to Amir-Shapira [46].

### Statistical analyses

Parameters were statistically tested by analyses of variance and comparisons of means were performed with a Duncan test (*P* < 0.05). Statistical analyses were performed with SPSS, version 22.0 (IBM, Armonk, NY, USA). Figures were prepared using the Origin 8.0 software (OriginLab, Hampton, MA, USA).

## Additional files

Additional file 1: **Figure S1.** Correlation of gene expression level between samples. (TIF 76 kb)

Additional file 2: **Table S1.** GO term enrichment analysis. (XLSX 28 kb)

Additional file 3: **Table S2.** Gene information in subclusters. (XLSX 50 kb)

Additional file 4: **Table S3.** KEGG pathway analysis. (XLSX 13 kb)

Additional file 5: **Table S4.** Real-time PCR primer pairs. (XLSX 51 kb)

## Abbreviations

APX: Ascorbate peroxidase
CAO: Chlorophyllide a oxygenase
CBR: Chlorophyll (ide) b reductase
Chl: Chlorophyll
CS: Chl synthase
DEGs: Differentially expressed genes
ETR: Relative electron transport rates
F0: Minimum fluorescence
Fe: Iron
Fm: Maximum fluorescence
Fv/Fm: Maximum quantum yield
GGPP: Geranylgeranyl pyrophosphate
GO: Gene ontology
KEGG: Kyoto encyclopedia of genes and genomes
Lhcs: Light-harvesting complexes
NCED: 9-cis-epoxycarotenoid dioxygenase
POD: Peroxidase
Pro: Proline
PSI: Photosystem I
PSII: Photosystem II
PSY: Phytoene synthase
qRT-PCR: Quantitative real-time PCR
β-OHase: β-carotene hydroxylase
RNA-Seq: RNA sequencing
SOD: Superoxide dismutase
VDE: Violaxanthin de-epoxidase
Y(II): Effective quantum yields
Y(NO): Quantum yield of non-regulated energy dissipation
Y(NPQ): Quantum yield of regulated energy dissipation
ZEP: Zeaxanthin epoxidase
