# Supplementary figures and images for: Transcriptional and physiological analyses of short-term Iron deficiency response in apple seedlings provide insight into the regulation involved in photosynthesis
Source: BMC Genomics. 2018 Jun 15;19:461. doi: 10.1186/s12864-018-4846-z (PMC6003109; doi:10.1186/s12864-018-4846-z)

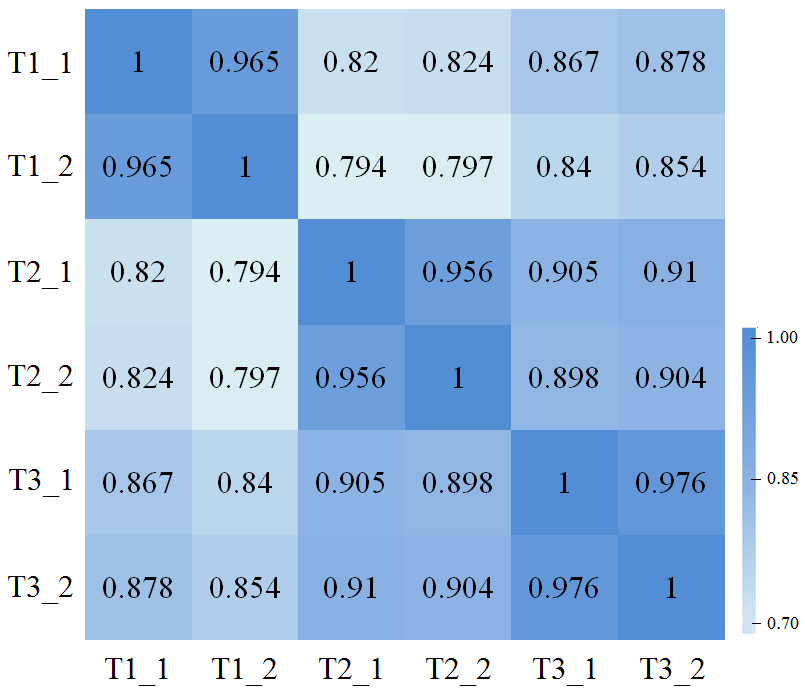

Supplement: Supplementary file 1 — Figure S1. Correlation of gene expression level between samples. (TIF 76 kb) [file 12864_2018_4846_MOESM1_ESM.tif]
